# Efficacy and Safety of the Two Ayurveda Drug Regimens in Uterine Fibroids: A Randomized Single-Blind Clinical Trial

**DOI:** 10.1155/2021/4325502

**Published:** 2021-06-28

**Authors:** K. P. K. R. Karunagoda, P. K. Perera, H. Senanayake, S. De Silva Weliange

**Affiliations:** ^1^Institute of Indigenous Medicine, University of Colombo, Colombo, Sri Lanka; ^2^Department of Obstetrics and Gynecology, University of Colombo, Colombo, Sri Lanka; ^3^Department of Community Medicine, University of Colombo, Colombo, Sri Lanka

## Abstract

This study aims to assess the efficacy and safety of two Ayurveda drug regimens for the treatment of uterine fibroids (UF) in a randomized single-blind clinical trial. 120 participants with UF (volume ≥ 2 cm^3^) were randomly allocated at a 1 : 1 : 1 ratio to 2 experimental groups and the control group. The 12-week intervention period was followed by 12-week follow-up. The primary efficacy endpoint was the change of the largest UF volume. The secondary efficacy endpoints were assessed by the pictorial bleeding assessment score (PBAC), UF symptoms, and quality of life score. The safety endpoints were changed in hepatic and renal safety parameters and patients experiencing adverse effects. Significant decrease was observed in the volume of UF in the arm II but not in arm I, while a significant increase was observed in the volume of the largest UF in the control group at 12^th^ week. The PBAC score remained stable in all groups. Further mean value of the intervention arms symptom severity subscale (SSS) was significantly reduced compared to the control arm. Health-related quality of life (HRQL) value improved in 12^th^ week of both experimental arms compared to baseline. Control arm HRQL value was reduced compared to baseline. The volume of the largest UF and both SSS and HRQL values remained stable within the follow-up period in the tested arms. The findings of this study demonstrated the safety and efficacy of selected two Ayurveda drug regimens in reducing the volume of UF and related symptoms and improving quality of life.

## 1. Introduction

Uterine fibroids are the most common reproductive tract tumor in the child-bearing age of women [1, 2]. Women with fibroids create considerable personal and social costs including diminished quality of life, disruption of usual activities and roles, lost work time associated with symptoms, and substantial healthcare expenditures [3]. Eventhough there are limited medical therapies, surgical and other invasive interventions still dominate in fibroid treatment. Many women are opposed to having a hysterectomy due to the undesirable comorbidities such as inpatient hospitalization, prolonged fever, transfusion, scarring, relatively long recovery time to presurgical levels of activities, and elimination of future pregnancies [4].

In last few decades, pharmacologic agents are used to provide relief for fibroid patients with mild symptoms, including combined oral contraceptives, progesterone (by oral, injection, or intrauterine device), and nonsteroidal anti-inflammatory drugs, antifibrinolytics, gonadotropin-releasing hormone agonists, selective estrogen or progesterone receptor modulator progestin, danazol, and aromatase inhibitors. Some of these medications are also useful in reducing tumor growth [5]. But still, there is no any gold stranded treatment option being developed for this common condition. In this context, various complementary and alternative medicine treatments have been administered for the uterine fibroids amelioration process including the Sri Lankan Ayurveda which is the most popular complementary medicine in Sri Lanka. But, further research studies are still an immense requirement for these treatment modalities.

Sri Lanka Ayurveda is a mixture of Ayurveda and indigenous medicine. Sri Lanka developed its own Ayurveda system based on a series of prescriptions handed down from generation to generation over a period of 3,000 years. Uterine fibroids have been treated by Ayurveda medicine regimens in Sri Lanka. It was noticed that a considerable number of patients search Ayurveda treatment for this condition to prevent surgery and preserve fertility [6]. The treatment regimens selected for this study were freely available in Sri Lanka and extensively used in the treatment of uterine fibroids without any adverse reactions and proven effectiveness clinically [7]. However, the effectiveness and safety of selected two treatment regimens in uterine fibroid patients have not been explored by the scientific clinical trials. Therefore, this research was designed as a randomized clinical trial which has been considered as a gold standard from the clinical research paradigm. The study aimed to compare the efficacy and safety of selected two Ayurveda drug regimens in the treatment of uterine fibroids.

## 2. Methodology

### 2.1. Study Design and Oversight

This study was a randomized single-blind clinical trial conducted in gynecology clinic of National Ayurveda Teaching Hospital, Borella, Sri Lanka during years 2018–2020. The ethics approval for this clinical trial was obtained from the Research Approval Committee of the Faculty of Graduate Studies in the University of Colombo and the Ethics Review Committee of Institute of Indigenous Medicine in University of Colombo, Sri Lanka. The trial was registered in ISRCTN registry (trial number: ISRCTN16108738). The study was conducted adhering to good clinical practice guidelines. Written informed consent was obtained from each participant before conducting the trial. The participants were given sufficient time to ask questions and decide whether they wish to participate in this study or not.

### 2.2. Study Population

Sample size was calculated with the main outcome parameter as reduction in the uterine fibroid size (each arm, 30 + loss to follow-up 10) [8]. A total 120 women aged between 18 and 50 years with at least one fibroid ≥2 cm^3^ in volume as assessed by ultrasonography were included for the study. The main exclusion criteria were menorrhagia (score ≥ 100 by PBAC), serum hemoglobin level less than 11 g/dL, having used any steroid hormonal therapy for a minimum of 03 months prior, pregnancy, lactating females, menopausal women, presence of condition other than fibroids, contributing to dysmenorrhea or pressure symptoms, and presence of any severe medical or psychological condition that, in the opinion of the investigator, would compromise the patient's safe participation.

### 2.3. Randomization and Intervention

Participants were randomly allocated at a 1 : 1 : 1 ratio to either the two experimental groups or to the control group (40 per each arm) by block randomization generated using an online program. The two experimental groups (I and II) received two Ayurveda drug regimens (Table 1), and the control group was kept as observation without treatment. Selected 11 Ayurveda products purchased from Sri Lanka Ayurvedic Drugs Corporation were included in Sri Lankan Ayurveda Pharmacopoeia [9, 10].

The duration of intervention was 12 weeks followed by a 12-week follow-up period. All the cases were advised to visit clinic once in two weeks for monitoring. The drugs for the next two weeks were issued for the experimental group. The duration of interventions, drugs, and treatment procedures are given in Table 1.

### 2.4. Efficacy Assessment

The primary efficacy endpoint was the change of the largest uterine fibroid volume at the end of the treatment. The secondary efficacy endpoints were assessed by the pictorial bleeding assessment score scale, the uterine fibroid symptoms, and the quality of life scale.

Fibroid volume was measured with the help of the GE Voluson P6 ultrasound machine 4C-RS convex probe or RICS-9A endocavitary probe by an expert ultrasonographer, who was blind to this trial. Volume of the largest fibroid was measured by transvaginal ultrasound scan at the screening, the posttreatment (12^th^ week), and at the follow-up (24^th^ week) by applying the prolate ellipsoid method (formula *V* = 0.5233 (*D*1 × *D*2 × *D*3) [11].

### 2.5. Assessment of Uterine Bleeding

Cases of menorrhagia were excluded from this study. Since the selected drug regimens were not tested for menstrual behaviors. Patients were advised to record their bleeding pattern by the pictorial bleeding assessment chart (PBAC) at the screening and after the treatment.

### 2.6. Assessment on Symptom Severity and Quality of Life (QOL)

Symptom severity and their impact on health-related quality of life (HRQL) were assessed by a Uterine Fibroid Symptom and Health-Related Quality of Life Questionnaire (UFS-QOL) already translated by a scientific method and validated in a small population at the same study center [12]. The questionnaire consists of an 8-item symptom severity scale and 29 HRQL questions, which comprise 06 subscales including concern, activities, energy/mood, control and self-consciousness.

### 2.7. Safety Analysis

Each patient underwent the hematological investigations (full blood count (FBC), aspartate aminotransferase (ALT), alanine aminotransferase (AST), serum creatinine, glomerular filtration rate (GFR), and urine full report (UFR)) before and after the treatment. The vital signs were measured and recorded in a patient diary at each visit. The safety endpoints were considered as the number and proportion of patients withdrawing from treatment early for safety reasons, changes in hepatic and renal safety parameters, and the number and proportion of patients experiencing adverse effects.

### 2.8. Statistical Analysis

Statistical analysis was performed using the SPSS statistical package program (ver. 22.0), and the level of significance was established at *α* = 0.05. Descriptive analysis for categorical variables was expressed as numbers and proportion. The continuous data were assessed for normalcy using QQ plots and the Kolmogorov–Smirnov test. Continuous data were described using mean and standard deviation for normally distributed variables, while skewed data were described using IQR. Between-group comparisons were carried out by analysis of variance (ANOVA) for normal data, while for nonnormal data, the Kruskal–Wallis test was used. For before and after data comparisons, the paired *t*-test and the related samples Wilcoxon sign rank test were used for normally distributed and nonnormal distrusted data, respectively. Similarly, for categorical variables, the chi-square test and Mac Nemar chi-square test were used, respectively. Intention to treat analysis was performed for all efficacy outcomes and safety outcomes.

## 3. Results

### 3.1. Consort Flow Diagram of Group's Enrollment, Allocation, Follow-Up, and Analysis

**Figure 1:**
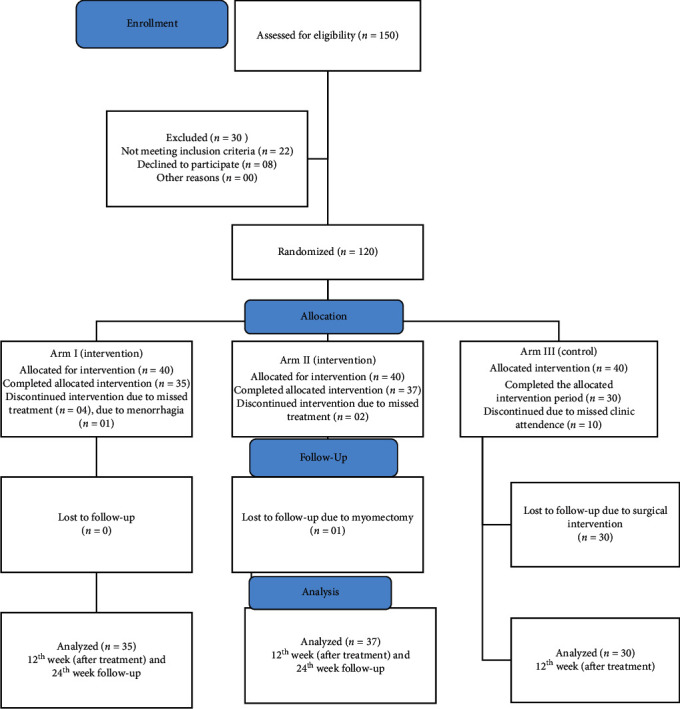
Consort diagram of the study.

150 participants who were referred to the Gynecology Unit of National Ayurveda Teaching Hospital were assessed for eligibility, and possible patients who fulfilled the inclusion criteria were enrolled to this study (Figure 1). 22 cases did not met with the inclusion criteria and 8 cases declined considering the study perspective after discussing the information provided by the investigator. Excluded cases were continued with the regular clinical management. Included 120 cases were randomly divided into three arms. The total study period of the randomized clinical trial was completed by 102 women: 35 (87.5%) cases from arm I, 37 (92.5%) from arm II, and 30 cases (75%) from control (arm III). Within the study period with follow-up of 12 weeks, 04 cases left without completing the programme in arm I due to not regularly attending the clinics and 01 case left the trial due to menorrhagia. In arm II, 02 cases left without completing the trial due to not regularly attending the clinics and 01 case underwent myomectomy due to no improvement of the symptoms. All the cases recruited to arms I and II completed the follow-up period (12^th^–24^th^ week). In control (arm III), all the cases visited the regular clinics in first 12 weeks, but the follow-up period (12^th^–24^th^ week) was not completed due to their planned myomectomy.

### 3.2. Baseline Characteristics of the Study Sample

Baseline characteristics of the 102 participants who completed the study and who entered to three arms were very similar characters such as most women were in their late thirties or early forties married stage, employed, and Buddhist. The *P* value shows a nonsignificant difference (*P* > 0.05) between the arms (Table 2).

### 3.3. Efficacy Outcomes of Three Arms

As given in Table 3, there was no significant reduction in the volume of the fibroids in the arm I (MD: 13.81, *P*=0.312) and significant reduction in arm II (MD: 0.38, *P*=0.001), and there was a significant increase in the size of fibroids in the control group (Arm III MD: −45.43, *P*=0.001). Mean difference of fibroid volume in two experimental arms from 12^th^ week to 24^th^ week (follow up) was nonsignificantly increased in mean volume by 0.22 cm^3^ in Arm I and 3.03 cm^3^ in Arm II.

The difference of the PBAC score between groups at 12^th^ week was nonsignificant. The symptom severity mean value differences from baseline to 12^th^ week of all the arms were significant (*P* < 0.05). The experimental arms mean values were not changed at the follow-up compared to after treatment. HRQL subscale items mean values of experimental arms were increased in 12^th^ week compared to the baseline (*P* < 0.05). There was no significant difference shown in HRQL total score in 24^th^ week when comparing with the posttreatment values.

### 3.4. Safety Outcomes of Three Arms

The mean values of serum AST, ALT, and serum creatinine at baseline and the 12^th^ week differences were not significant in all the groups. Important parameters of serum creatinine, GFR assessment, FBC, and UFR at baseline and the 12^th^ week are given table. The urobilinogen, bile pigments, WBC, platelet count, and hematocrit mean values remained stable in the study period.

## 4. Discussion

This was the first study assessing the 6-month clinical outcome of selected 02 Ayurveda drug regimens for the management UF. In general, guidelines for methodologies on research and evaluation of traditional medicine also recommend the use of both herbal medicine and traditional procedures-based therapies together with the treatment. Because they believe that the successful treatment is often the consequence of both types of treatment acting synergistically [13].

This randomized single-blind clinical trial was completed with 102 participants with UF (Figure 1). The mean age range between 37 and 39 was reported in this study following the normal age range reported by the previous studies [14]. The clinical trial participants were also reflecting the religious diversity of Sri Lanka. Furthermore, occupation and education showed the normal distribution pattern of urban women of Sri Lanka. The mean BMI of arms I, II, and III was 23.61 (3.39), 23.40 (4.11), and 23.29 (4.27), respectively. Comparison between three arms on Hb%, mean PBAC score, the volume of the largest fibroid, and QOL score also showed nonsignificant (*P* > 0.05) variation. Demographic data were not significantly variable in the three arms (Table 2). Therefore, a comparable study can be made in this research' findings where most of such other studies were unable to perform [15].

The results indicated a reduction of the fibroid volume at 12^th^ week in both experimental groups (arm I mean difference: 13.81 cm^3^; arm II mean difference: 0.38 cm^3^) which was significant in arm II (*P* > 0.05) (Table 3). In the control arm, the mean fibroid volume significantly increased (*P* > 0.05). These findings were in agreement with the results reported in previous case series and case studies conducted on uterine fibroid volume reduction with several Ayurveda drug regimens [16–20]. The mean UF volume measurements of both intervention arms in the follow-up period did not report a significant change from 12^th^ week (*P* > 0.05). When comparing both the arms, arm II showed a slight increase of the volume than arm I at the end of 24^th^ week of the follow up. Posttreatment fibroid regrowth reported by some of the medical management includes asoprisnil, [21] and stable UF volume in 3 months follow-up after three months treatment with ulipristal acetate (UPA) [22]. But still, no published data are available from Ayurveda drug regimens to compare the results. In the follow-up period (12^th^–24^th^ week), results of the arms I and II could not be compared with the control (arm III). In the follow-up period (12^th^ week to 24^th^ week), comparing the effects of two Ayurveda treatment regimens (arms I and II) indicated that the UF volume was not significantly changed even after cessation of the treatment (Table 3).

Research studies have had to define their own criteria for treatment success, and the PBAC is one [23]. It is commonly understood that the PBAC score of 100 or more is heavy menstrual bleeding, 99–02 considered normal bleeding, and PBAC score less than 02 is amenorrhea. In this study, the menstruation pattern was not changed in either group after the treatment; furthermore, 07 cases reported menorrhagia in arm I and 04 cases in arm II which might get some effects by the treatment (Table 4). But these reported changes were not taken seriously as they were controlled without any added intervention or discontinuation of the study intervention. Furthermore, all the cases remained normal hemoglobin levels throughout the period which supported the augment made. Anyhow, some relations of menstrual bleeding with the treatment may be proved by a large-scale clinical study. Cases were not reported with amenorrhea by this study where most of the medical managements, UPA [24], GnRH, agonist selective progesterone receptor modulators (SPRMs) of the disease were faced.

Studies explored that, with the increasing availability of noninvasive therapies to hysterectomy, it will be important to assess symptom reduction of uterine fibroids of patients who choose these treatment options [25]. The patients with symptomatic uterine fibroids treated by Ayurveda drug regimen experienced significant alleviation of fibroid-related symptoms (by SSS) and increased in HR-QOL. Baseline mean values of SSS remained nonsignificant between three arms (*P*=0.615). This was a moderate level of severity when the highest score indicates greater symptom severity. This could be a specific factor of following Ayurveda treatment by the study population as they can bear up the disease-related difficulties up to some extent till the treatment initiates it action. In posttreatment, assessment mean values of SSS were reduced in experimental arms, while the increased mean value was shown in control. Then, the SSS values of baseline and after treatment were compared by the paired *t*-test. Both the experimental arms mean values of SSS at 12^th^ week (after treatment) were reduced (symptoms reduced), while in control, SSS was improved (*P* < 0.05). This result proved that there was an effect on Ayurveda treatment on controlling symptoms related to the uterine fibroid. Considerable symptoms at the enrollment and a substantial decrease in mean symptom levels after intervention appear to be a clinical amelioration of the disease. The UFS-QOL has a second scale that measures six dimensions of health-related QOL (HR-QOL). In contrast to the SSS, on this 100-point scale, a lower number indicated improved QOL. These QOL impacts have included fatigue, self-consciousness, weight gain, interference with physical activities, and interference with daily and social activities and effect on relationships with partners and with family and friends, impaired ability to take care of home or children, and missed workdays [26]. Two intervention arms reported a significant improvement in HRQL after treatment compared to baseline. The control arm value was significantly reduced. Previous studies claimed that the clinical efficacy of alternative uterine sparing treatments for UF cannot be fully described by objective measurements, such as changes in UF volume, because symptom alleviation is highly subjective [27]. At this point, the achieved positive clinical outcome by the UFS-QOL can be regarded as a marker for clinical success of the selected drug regimens. Follow-up values of the UFS-QOL (both SSS and HRQL scales) were not changed significantly and persist near to the posttreatment stage (Table 4). The fact that the UFS-QOL score changes follow volume reduction behavior occurred (changed significantly at posttreatment and then static at the follow-up) argues against this being a result of bias or a placebo effect.

Safety biomarkers are important tools in clinical trials as they are measurable indicators of normal biological processes or biological responses to a therapeutic intervention. Eventhough herbal remedies and Ayurveda treatments are believed to be safe, it is highly recommended to carry safety studies in every clinical trial. The results of this study show the efficacy and the safety of two regimens. Serum AST, ALT, creatinine, GFR, and parameters of full blood count and urine full report remained unchanged and within the normal range (Table 5). None of the treated patients had treatment-related serious adverse effects. There were no major adverse effects and clinical implications noted in this conducted trial. There were no deaths, life-threatening events, or unintended second procedures reported. A single participant continued heavy menstruation in the second week after the treatment initiation, requiring switch to bleeding, controlling Ayurveda treatments by which cases were controlled in ten days. She was discontinued from the study. Another complaint of treatment emergent gastritis is at the initial stage, but it was suppressed by proper food habits. Hence, these two herbal treatment approaches may be a safety answer for short-term treatment of uterine fibroids where some studies could not be achieved. Most of other medical interventions were questionable in the safety aspect [28] when treating leiomyoma. Considering treatment approaches of the conducted trial, there were no safety implications raised.

It is clear that both the Ayurveda drug regimens demonstrated efficacy and safety in treating uterine fibroids. The results of this study may provide answers for the women with uterine fibroid who have pain and pressure effects, women who wish to retain the option of childbirth, women who wish to save their uterus, women who are not fit for surgical intervention, and women with infertility can take advantage of this type of treatment.

## 5. Conclusion

In conclusion, the findings of this study demonstrated the efficacy of selected two Ayurveda drug regimens in fibroid shrinking, reducing fibroid-related symptoms, and improving quality of life. It was confirmed that the two drug regimens were safe for use in uterine fibroid treatment. Furthermore, we suggest that multicentered randomized controlled trials are needed to confirm our findings in future.

## Figures and Tables

**Table 1:** Procedure of intervention.

Period	No.	Arm I	Arm II	Arm III
02 weeks	1	Panchamoolilaghudrakshadi decoction, 30 ml, bd		No intervention (control arm)
	2	Chandraprabha Vati, 2 pills (500 mg × 2), bd		
	3	Manibadra Choorna, 05 g powder, at night		
03^rd^–12^th^ week	4	Thiplagugul decoction, 30 ml, bd	Punarnavashtaka decoction, 30 ml, bd	
	5	Panchatiktagritaguggul, 2 pills (500 mg × 2), bd	Kanchanaragugulu, 2 pills (500 mg × 2), bd	
	6	Krishna Jeeraka Choorna 5 g powder, bd	Satapushpa Choorna 5 g powder, bd	
	7	Sharshapadi oil—external application on lower abdomen for 07 days after each menstruation	Nirgundyadi oil—external application on lower abdomen for 07 days after each menstruation	
13^th^–24^th^ week		No intervention (follow-up)		

**Table 2:** Baseline characteristics of the study sample.

Characteristics	Arm I (*n* = 35)	Arm II (*n* = 37)	Arm III (*n* = 30)	*P* value^*∗∗*^
Age (y), mean (SD)	38.66 (6.54)	37.11 (5.82)	39.59 (6.33)	0.258
Marital status, *n* (%)				0.708
Married	26 (74.3)	27 (71.1)	22 (75.9)	
Single	07 (20.0)	10 (26.3)	7 (24.1)	
Widowed	02 (5.7)	01 (2.6)	0 (0.0)	

Occupation, *n* (%)				0.851
Work outside the home	26 (74.3)	26 (68.4)	21 (72.4)	
Homemaker	09 (25.7)	12 (31.6)	08 (27.6)	

BMI (kg/m^2^), mean (SD)	23.61 (3.39)	23.40 (4.11)	23.29 (4.27)	
Hemoglobin (%), g/dL	11.83 (0.80)	12.20 (1.06)	12.12 (0.81)	0.185
PBAC score, mean (SD)	54.17 (31.31)	50.86 (25.81)	62.23 (27.71)	0.262

Arm I and Arm II, experimental groups; Arm III, control group; BMI, body mass index; PBAC, pictorial bleeding assessment chart. ^*∗∗*^Significant level of *P* < 0.05 by ANOVA.

**Table 3:** Fibroid volume change in 12^th^ week and 24^th^ week of three arms.

Fibroid volume (cm^3^)	Arm I	Arm II	Arm III	*P* value^*∗∗*^
Baseline	*n* = 35	*n* = 37	*n* = 30	
Baseline (mean ± SD)	82.03 (173.08)	86.67 (161.20)	66.63 (151.14)	0.525

12^th^ week (after treatment)	*n* = 35	*n* = 37	*n* = 30	
12^th^ week (mean ± SD)	68.22 (142.11)	86.29 (237.19)	112.06 (240.52)	0.322
Mean difference from baseline to 12^th^ week (MD)	13.81	0.38	−45.43	
*P* value^*∗*^	0.312	0.001	0.001	

Follow-up period				
24^th^ week (follow-up)	*n* = 35	*n* = 37	*n* = 00	
24^th^ week (mean ± SD)	68.45 (137.19)	89.32 (221.58)	—	0.813^#^
Mean difference from 12^th^ week to 24^th^ week (MD)	−0.22	−3.03	—	
*P* value^*∗*^	0.145	0.780	—	

Arm I and Arm II, experimental groups; Arm III, control group; SD, standard deviation; MD, mean difference. ^*∗∗*^Significant level of *P* < 0.05, based on the related samples Wilcoxon sign rank test (before and after within group). ^*∗∗*^Significant level of *P* < 0.05, based on the Kruskal–Wallis value test (between group). ^#^Between Arm I and Arm II.

**Table 4:** The secondary efficacy endpoints changes in the 12^th^ week and 24^th^ week.

Variable	Arm I (*n* = 35)	Arm II (*n* = 37)	Arm III (*n* = 30)	*P* value
PBAC score MD (% change)	1.02 (44.31)	−10.97 (−33.73)	−2.90 (−14.44)	
PBAC at 12^th^ week				
≥100 (menorrhagia)	01 (02.9)	04 (10.8)	02 (06.7)	0.410^*∗*^
99–02 (normal flow)	34 (97.1)	33 (89.2)	28 (93.3)	
<02 (amenorrhea)	00 (0.0)	00 (0.0)	00 (0.0)	

UFS-QOL-symptom severity^*α*^				
Baseline (mean ± SD)	33.21 (15.91)	32.26 (16.52)	30.72 (14.21)	0.815^*∗∗*^
12^th^ week (mean ± SD)	25.71 (16.45)	22.63 (12.74)	34.16 (14.77)	0.006^*∗∗*^
*P* value^*∗∗∗*^ (baseline to 12^th^ week)	0.001	0.001	0.006	
24^th^ week mean difference from 12^th^ week (MD ± SD)	0.00 (2.00)	0.00 (0.00)	—	
*P* value^*∗∗∗*^ (12^th^–24^th^ week)	1.00	0.05	—	

UFS-QOL-HRQL^*α*^^*α*^				
Baseline (mean ± SD)	51.45 (16.98)	58.57 (17.48)	59.13 (15.51)	0.112^*∗∗*^
12^th^ week (mean ± SD)	57.73 (17.37)	64.63 (17.63)	55.35 (16.47)	0.081^*∗∗*^
*P* value^*∗∗∗*^ (baseline to 12^th^ week)	0.005	0.035	0.010	
24^th^ week (mean ± SD)	57.53 (17.14)	64.60 (17.14)	—	
*P* value^*∗∗∗*^ (12^th^–24^th^ week)	0.160	0.786	—	

Arm I and Arm II, experimental groups; Arm III, control group; PBAC, pictorial blood loss assessment chart; score value ≥ 100, menorrhagia; 99–02, normal flow; <02, amenorrhea; HRQL, health-related quality of life; UFS-QOL, Uterine Fibroid Symptom and Health-Related Quality of Life Questionnaire; SD, standard deviation; MD, mean difference. ^*∗*^Significant level of *P* < 0.05, based on the chi-square test (between groups). ^*∗∗*^Significant level of *P* < 0.05, based on ANOVA (between groups). ^*∗∗∗*^Significant level of *P* < 0.05, based on the paired *t*-test (before and after within groups). ^*α*^Score range from 0 to 100, a higher score indicates greater symptom severity; ^*α*^^*α*^score range from 0 to 100, higher scores indicate better HRQL.

**Table 5:** The safety outcomes of three arms.

Variable	Arm I (*n* = 35)	Arm II (*n* = 37)	Arm III (*n* = 30)
AST (IU/L)			
Baseline	20.47 (9.45)	18.38 (6.39)	18.77 (8.15)
12^th^ week	20.13 (7.48)	18.87 (5.43)	18.40 (8.17)
*P* value	0.78	0.63	0.31

ALT (IU/L)			
Baseline	20.63 (10.30)	20.09 (9.01)	19.86 (11.19)
12^th^ week	20.90 (9.8)	19.38 (9.10)	19.71 (11.12)
*P* value	0.84	0.54	0.35

Creatinine (mg/dl)			
Baseline	0.68 (0.10)	0.63 (0.10)	0.69 (0.14)
12^th^ week	2.1 (8.67)	2.04 (8.44)	0.69 (0.14)
*P* value	0.33	0.31	0.20

GFR (mL/min/1.73 m^2^)			
Baseline	>90	>90	>90
12th week	>90	>90	>90

Urobilinogen (mg/dL)			
Baseline	Normal	Normal	Normal
12^th^ week	Normal	Normal	Normal

Bile pigments			
Baseline	Nil	Nil	Nil
12^th^ week	Nil	Nil	Nil

WBC^*∗∗*^ (10^9/L)			
Baseline	28.34 (107.48)	28.47 (121.42)	24.19 (101.11)
12^th^ week	28.64	28.25	26.30

PLT^*∗∗*^ (10^9/L)			
Before	343 .57 (80.30)	339.41 (89.87)	331.63 (86.73)
12^th^ week	354.71 (69.74)	342.22 (77.77)	339.03 (88.78)

Hematocrit (%)			
Before	34.52 (4.89)	37.48 (5.17)	36.29 (4.34)
12^th^ week	36.65 (9.67)	37.47 (8.63)	36.38 (4.26)

Values are presented as mean ± standard deviation. AST, aspartate transaminase; ALT, alanine aminotransferase; GFR, glomerular filtration rate; TB, total bilirubin; WBC, white blood cells; PLT, platelet. ^*∗*^Based on urine full report. ^*∗∗*^Based on full blood count. ^*∗*^Significant level of *P* < 0.05 by the paired *t*-test.

## Data Availability

The data used to support the findings of this study are available from the corresponding author upon request.
